# Exploring the alterations and function of skin microbiome mediated by ionizing radiation injury

**DOI:** 10.3389/fcimb.2022.1029592

**Published:** 2022-11-14

**Authors:** Biao Huang, Lu An, Wenxing Su, Tao Yan, Haifang Zhang, Dao-Jiang Yu

**Affiliations:** ^1^ Department of Plastic and Burn Surgery, The Second Affiliated Hospital of Chengdu Medical College, China National Nuclear Corporation 416 Hospital, Chengdu, China; ^2^ Department of Clinical Medicine, Chengdu Medical College, Chengdu, China; ^3^ Transformation Center of Radiological Medicine, The Second Affiliated Hospital of Soochow University, Suzhou, China; ^4^ West China School of Basic Medical Sciences and Forensic Medicine, Sichuan University, Chengdu, China; ^5^ Department of Clinical Laboratory, The Second Affiliated Hospital of Soochow University, Suzhou, China

**Keywords:** skin microbiome, radiation-induced skin injury, 16S rRNA, radiation protection, microbial metabolism

## Abstract

**Background:**

Radiation-induced skin injury (RISI) is still the most common and severe side effect of radiotherapy. The role of the skin’s microbial barrier in the pathogenesis and progression of RISI needs to be fully investigated.

**Methods:**

This study aimed to explore the alterations in and functions of the skin microbiota in RISI. We applied the unculturable approach to characterize the cutaneous microbiomes of a radiation-induced animal model by sequencing the V1–V3 regions of the 16S ribosomal RNA (rRNA) gene. Combined with the downloaded clinical data of patients, a comprehensive analysis was performed to identify potential radioprotective species and metabolic pathways.

**Results:**

There were no significant differences in the alpha diversity indices (Sobs, Shannon, Simpson, Ace, and Chao) between the acute radiation injury and control groups. Phylum-level analysis of the RISI microbiomes exhibited significant predominance of Firmicutes (mean abundance = 67%, corrected *p* = 0.0035). The high abundance of Firmicutes was significantly associated with rapid healing of RISI (average relative abundance = 52%; Kruskal–Wallis: *p* = 5.7E−4). Among its members, *Streptococcus*, *Staphylococcus*, *Acetivibrio ethanolgignens group*, *Peptostreptococcus*, *Anaerofilum*, and *UCG-002* [linear discriminant analysis (LDA) > 3, *p* < 0.05] were identified as the core genera of Firmicutes. In addition, Lachnosiraceae and *Lactobacillus* occupied an important position in the interaction network (*r* > 0.6, *p* < 0.05). The differential metabolic pathways of RISI were mainly associated with carbohydrate metabolism (butanoate and propanoate metabolism), amino acid metabolism (tryptophan and histidine metabolism), energy metabolism, and lipid metabolism (fatty acid degradation and biosynthesis).

**Conclusion:**

This study provides new insights into the potential mechanism and skin microbial changes in the progression of RISI. The overwhelming predominance of members of Firmicutes, including Streptococcaceae, Staphylococcaceae, Lachnospiraceae, and *Lactobacillus*, is potentially related to rapid healing of RISI. The microbiota–metabolite axis plays a critical role in RISI and provides promising therapeutic targets for the treatment of adverse side effects.

## Introduction

Radiation-induced skin injury (RISI) is defined as cutaneous and includes deep tissue damage (Akh et al., 2022). It usually occurs following nuclear accidents, occupational exposure, and radiation therapy (Drozdovitch et al., 2022). According to research statistics, nearly 50% of patients with cancer receive radiotherapy, and 95% of them develop varying degrees of skin damage impacted by their age, physical condition, skin type, and the location of the tumor (Steinert et al., 2003). RISI is an irreversible and progressive condition that seriously deteriorates patients’ quality of life, even leading to the termination of therapy. On the whole, RISI is of two types: acute and chronic. Acute RISI includes dry and wet desquamation, skin necrosis, ulcers, and bleeding. Chronic RISI covers chronic ulcers, radiation-induced keratosis, telangiectasias, fibrosis, and skin cancer. RISI is a common and dose-limiting reaction. Following a cumulative radiation dose exceeding 10 Gy, the exposed skin often develops an intense local inflammatory reaction within 2 days to 1 week. The reaction peaks at 48 h, then subsides, only to be followed by a second phase of intense erythema with edema and vesiculation beginning 1 week after exposure and lasting up to 1 month. Erosions, pustule, and ulcerations may also develop with secondary infection. The mechanism of RISI is mainly related to skin cell senescence, fibrosis, vascular injury, radiation-induced reactive oxygen species (ROS) damage, and other signaling pathways (Wei et al., 2021; Yadav et al., 2022). Multiple therapeutic methods such as physical therapy, external dressing, and surgery have not been fully successful in the treatment of RISI and the prevention of damage to the adjacent tissue (Rosenthal et al., 2019).

As the largest organ of the human body, the skin structure forms a protective barrier against external invasion (Persinal-Medina et al., 2022). It is well known that the skin barrier functions as a microbial barrier, physical barrier, chemical barrier, and an immune barrier. Based on their integral collaboration, these barriers maintain the metabolic and immune homeostasis of the human skin (Celebi Sozener et al., 2022). As the outermost barrier against the external environment, the unique ecosystem constituted by colonizing microbiota has gradually been found to play an important role in the occurrence and progression of various inflammatory skin diseases. Factors affecting skin microbial colonization include lifestyle, systemic host factors, and environmental factors such as ionizing radiation damage (Luna, 2020). Although the skin microbiota has been intensely investigated regarding its significance in several diseases (atopic dermatitis, psoriasis, diabetic foot, and burns) in the past few years, research has been mainly based on the culture method (Alam et al., 2022; Durand et al., 2022). The role of skin microbiomes in the pathogenesis and prognosis of RISI remains to be fully studied, and the use of the unculturable approach helps provide a holistic view of the entire community (Wensel et al., 2022).

Our study provides insights into the alterations of the microbial barrier in RISI and identifies the core bacteria that may serve as potential targets for protection from and treatment of RISI.

## Materials and methods

### Ethics statement

The protocols for experiments involving animals were approved by the Animal Experimentation Ethics Committee at China National Nuclear Corporation 416 Hospital (Chengdu, China; reference no. SYXK2020-196).

### Animal model construction

A total of 29 male Sprague–Dawley rats (7–8 weeks old) were purchased from the Shanghai SLAC Laboratory Animal Co., Ltd. (Shanghai, China). These animals were housed in a pathogen-free environment at the facilities of the Medical College of Soochow and provided standard chow and water *ad libitum*.

Before irradiation, we collected skin samples from the gluteal region of 29 rats, which represents the control site. Subsequently, irradiated skin samples from the same site were taken as the treatment group.

For irradiation, the rats were anesthetized with an intraperitoneal injection of 10% chloral hydrate (10 mg/kg), and the hair on the gluteal region was shaved. The animals were immobilized using an adhesive tape on a plastic plate to minimize their motion during irradiation. A 3-cm-thick piece of lead was used to shield the animals and localize the radiation field (4 cm × 4.5 cm). The rats received a 40-Gy dose of radiation to the treatment area at a rate of 500 cGy/min using a 6-MeV electron beam accelerator (Clinac 2100EX; Varian Medical Systems, Palo Alto, CA, USA) for the construction of an acute skin radiation damage model. After irradiation, the exposed skin showed erythema and high temperature at around 10 days. After about 2 weeks, a large area of wet desquamation and superficial ulcers were formed, and the skin samples were collected at the same time.

### Sample collection

Defatted cotton swabs were pre-moistened in ST solution (0.15 M NaCl with 0.1% Tween-20). Thereafter, an area of 4 cm × 4.5 cm in the irradiated gluteal region was rubbed for 30 s for sample collection. A similar gluteal region before irradiation, which represents the control site, was swabbed to collect specimens from healthy subjects. Two to three swabs were saved per sample. After sampling, the cotton swab head was cut into a sterilized frozen tube, quickly frozen with liquid nitrogen, and stored at −80°C.

### DNA extraction and 16S rRNA gene sequencing

Skin sample preparation and 16S ribosomal RNA (rRNA) sequencing were conducted as previously described (Ellison et al., 2021; Olesen et al., 2022). Genomic DNA was extracted using Wizard^®^ Genomic DNA Purification Kit (Promega, Madison, WI, USA) according to the manufacturer’s protocol. Purified genomic DNA was quantified with a TBS-380 fluorometer (Turner BioSystems Inc., Sunnyvale, CA, USA). High-quality DNA [optical density (OD)_260/280_ = 1.8–2.0, >20 μg] was used for further analysis. The genome was sequenced using a combination of the PacBio RS II Single-Molecule Real-Time (SMRT) (PacBio, Menlo Park, CA, USA) and Illumina (San Diego, CA, USA) sequencing platforms. The Illumina data were used to evaluate the complexity of the genome. For Illumina sequencing, at least 1 μg genomic DNA was used for each strain to construct the sequencing library. The complete genome sequence was assembled using both the PacBio and Illumina reads. The original imaging data were converted into sequencing data *via* base calling, defined as the raw data or raw reads and saved as FASTQ files. These FASTQ files are the original data provided for users, which included the read sequences and quality information. A statistic of quality information was applied for quality trimming, by which low-quality data can be removed to obtain clean data. The reads were then assembled into a contig using Unicycler. The last circular step was checked and manually finished, generating a complete genome with seamless chromosomes and plasmids. Finally, error correction of the PacBio assembly results was performed using the Illumina reads with Pilon.

### Patient clinical data

Patient clinical data were from NCBI under accession no. PRJNA665254 (http://www.ncbi.nlm.nih.gov/bioproject/665254). Patients were included if they were newly diagnosed with grade 2 RISI. The exclusion criterion was systemic topical application of corticosteroids and antibiotics (Ramadan et al., 2021). The clinical characteristics of patients including age, sex, cancer type, and concomitant diseases are shown in **Table 1**.

**Table 1:** Clinicopathological characteristics of patients with radiation-induced dermatitis (RID).

Characteristics	RID cohort (*N* = 78)	Healthy cohort (*N* = 20)
Age (years)	49.42 ± 8.79	36.35 ± 13.46
Gender, *n* (%)		
Male	35 (45)	10 (50)
Female	43 (55)	10 (50)
Site of RISI, *n* (%)		
Chest	41 (53)	NA
Sacrum	13 (17)	NA
Pelvis	12 (15)	NA
Leg	3 (4)	NA
Head	5 (6)	NA
Neck	4 (5)	NA
Systemic disease, *n* (%)		
Diabetes	36 (46)	NA
Hypertension	21 (27)	NA
Clinical prognosis, *n* (%)		
Rapidly recovery	33(42)	NA
Delayed recovery	24(31)	NA
Chronic ulcer	21(27)	NA

RID, radiation-induced dermatitis; NA, not available.

### Bioinformatics analysis

The paired-end (PE) reads obtained with MiSeq sequencing were first spliced according to the overlap relationship, and the sequence quality was controlled and filtered at the same time. After distinguishing the samples, operational taxonomic unit (OTU) clustering analysis and species taxonomic analysis were carried out.

Non-repetitive sequences were extracted from optimized sequences to reduce the number of redundant computations (http://drive5.com/usearch/manual/dereplication.html). Single sequences without repetitions were removed, while the non-repetitive sequences (excluding single sequences) were clustered into OTUs according to 97% similarity, with the chimera removed in the clustering process to obtain the representative sequences of the OTUs.

Taxonomic analysis of the OTU representative sequences with 97% similarity was performed using the RDP classifier Bayesian algorithm. The community species composition of each sample was calculated at eight taxonomic levels: domain, kingdom, phylum, class, order, family, genus, and species. SILVA (http://www.arb-silva.de) and RDP (http://rdp.cme.msu.edu/) were used to compare databases.

Bacterial diversity was estimated using QIIME 2 for the entire dataset without subsampling (Callahan et al., 2016), and the alpha diversity was determined using two approaches: richness (number of observed species and Chao1 index) and evenness (Shannon diversity index) of communities. Unpaired Wilcoxon rank-sum test was used to assess the statistical significance between two groups. Principal coordinates analysis (PCoA) based on unweighted UniFrac distance matrix was used to calculate the similarities or differences of the community composition between two groups.

Based on the community abundance data in the sample, the Wilcoxon rank-sum test was used to detect species with significant differential abundance. The non-parametric factorial Kruskal–Wallis rank-sum test was used to determine the characteristics of the significant abundance differences and the group differences. Finally, linear discriminant analysis (LDA) effect size (LEfSe) was used to estimate the effect of the abundance of each component (species) (Segata et al., 2011).

Network analysis was conducted to obtain the coexistence relationship of species in the environment. The top 50 species based on the total abundance at the genus level were selected, and the Spearman’s correlation coefficients of these species were calculated. The size of the node represented species abundance, while the thickness of the line represented the correlation coefficient.

To predict the functional profile of cutaneous microbiota, the Greengenes database (v.13.8) at a 97% identity used a closed-reference script for OTU picking in QIIME (McDonald et al., 2012). The functional potential of cutaneous microbiomes was predicted using PICRUSt (http://picrust.github.io/picrust/) (Langille et al., 2013).

### Statistical analysis

Analyses of bacterial diversity, species differences, LEfSe, and PICRUSt were all performed and visualized using R software. A *p* < 0.05 or a false discovery rate (FDR) < 0.05 was considered as the statistical significance cutoff in all tests.

## Results

### Optimization of the 16S rRNA sequence

In total, the PE reads of 58 samples were input into QIIME 2 for merging, quality control and filtering, and correcting the direction of the sequences. The results revealed an optimized number of sequences of 3,842,947, optimized number of sequence bases of 1,614,133,683, and an average length of 420 bp.

### Species annotation and evaluation

**Figure 1:**
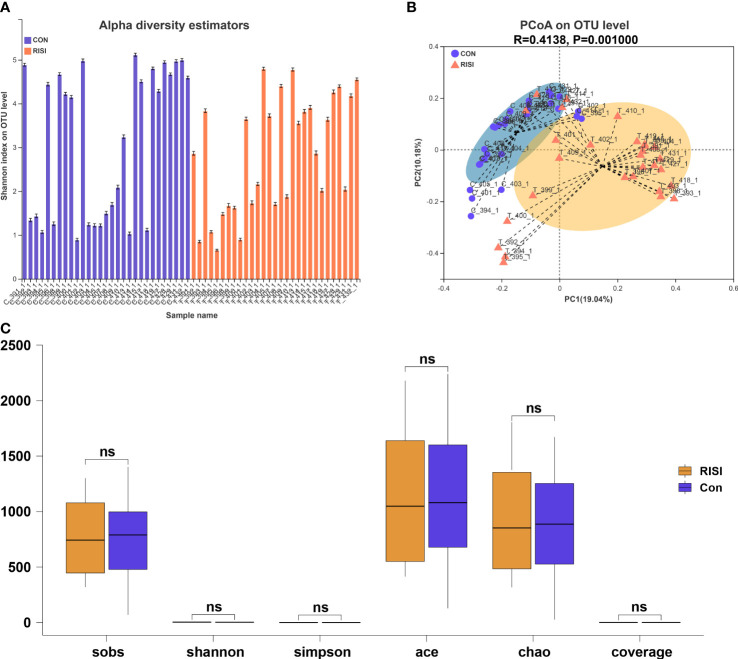
Diversity analysis of cutaneous microbiomes. **(A)** Alpha diversity bar charts between the radiation-induced skin injury (*RISI*) group and healthy controls (*CON*). **(B)** Beta diversity estimation. Principal coordinates analysis (PCoA) of RISI and CON based on unweighted UniFrac distance matrices. **(C)** Box plot of the comparison of the alpha diversity indices. ns: not sinificant.

According to the optimized sequence, the 16S rRNA reads were taxonomically assigned to 1 domain, 1 kingdom, 42 phyla, 108 classes, 254 orders, 424 families, 914 genera, 1,699 species, and 9,614 OTUs. There were no significant differences in the alpha diversity indices (Sobs, Shannon, Simpson, Ace, and Chao) between the acute radiation injury and control groups (*Q* > 0.05) (**Figures 1A, C**). In addition, the structure and composition of the bacterial communities significantly distinguished the classification of cutaneous microbiomes into RISI and healthy skin. PCoA based on unweighted UniFrac metrics sorted the samples into two particular clusters (*R* = 0.4138, *p* = 0.001) (**Figure 1B**).

### Composition differences between RISI and normal skin

**Figure 2:**
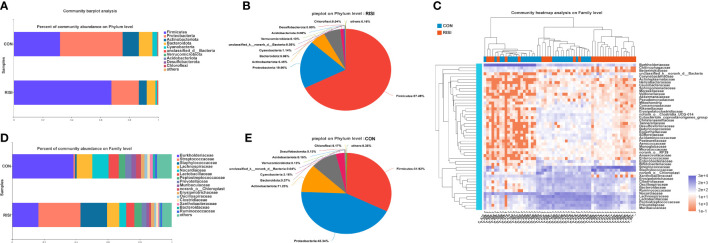
Species composition analysis. **(A)** Phylum-level analysis of the skin microbiota. *Bar charts* illustrate the relative proportions of dominant phyla across all study groups. **(B)** Pie chart illustrating the proportions of species at the phylum level in healthy skin. **(C)** Community heatmap. **(D)** Family-level analysis of the skin microbiota using bar charts. **(E)** Pie chart illustrating the proportions of species at the phylum level in radiation-induced skin injury (RISI).

In general, acute radiation injury-induced skin microbiome alterations showed remarkable differences in certain bacterial taxa at several classification levels compared with the healthy control. Phylum-level analyses of the RISI microbiomes exhibited alternately significant predominance of Firmicutes (mean abundance = 67%, corrected *p* = 0.0035) (**Figures 2A, B, E**). In contrast, the proportions of Proteobacteria (mean abundance = 19%, corrected *p* = 0.0029), Bacteroidetes (mean abundance = 6%, corrected *p* = 0.02), and Actinobacteria (mean abundance = 5%, corrected *p* = 0.0029) decreased significantly in RISI. In addition, Verrucomicrobia (mean abundance = 0.1%) showed a relatively constant proportion. Family-level analyses revealed that the abundances of Streptococcaceae (mean abundance = 26%, corrected *p* = 4.435E−5) (**Figures 2C, D**) and Staphylococcaceae (mean abundance = 17%, corrected *p* = 0.0004) significantly increased in RISI. In contrast, the proportions of Burkholderiaceae (mean abundance = 16%, corrected *p* = 0.0058), and Nocardiaceae (mean abundance = 4%, corrected *p* = 0.0007) significantly decreased in RISI.

### Differential species analysis of RISI

**Figure 3:**
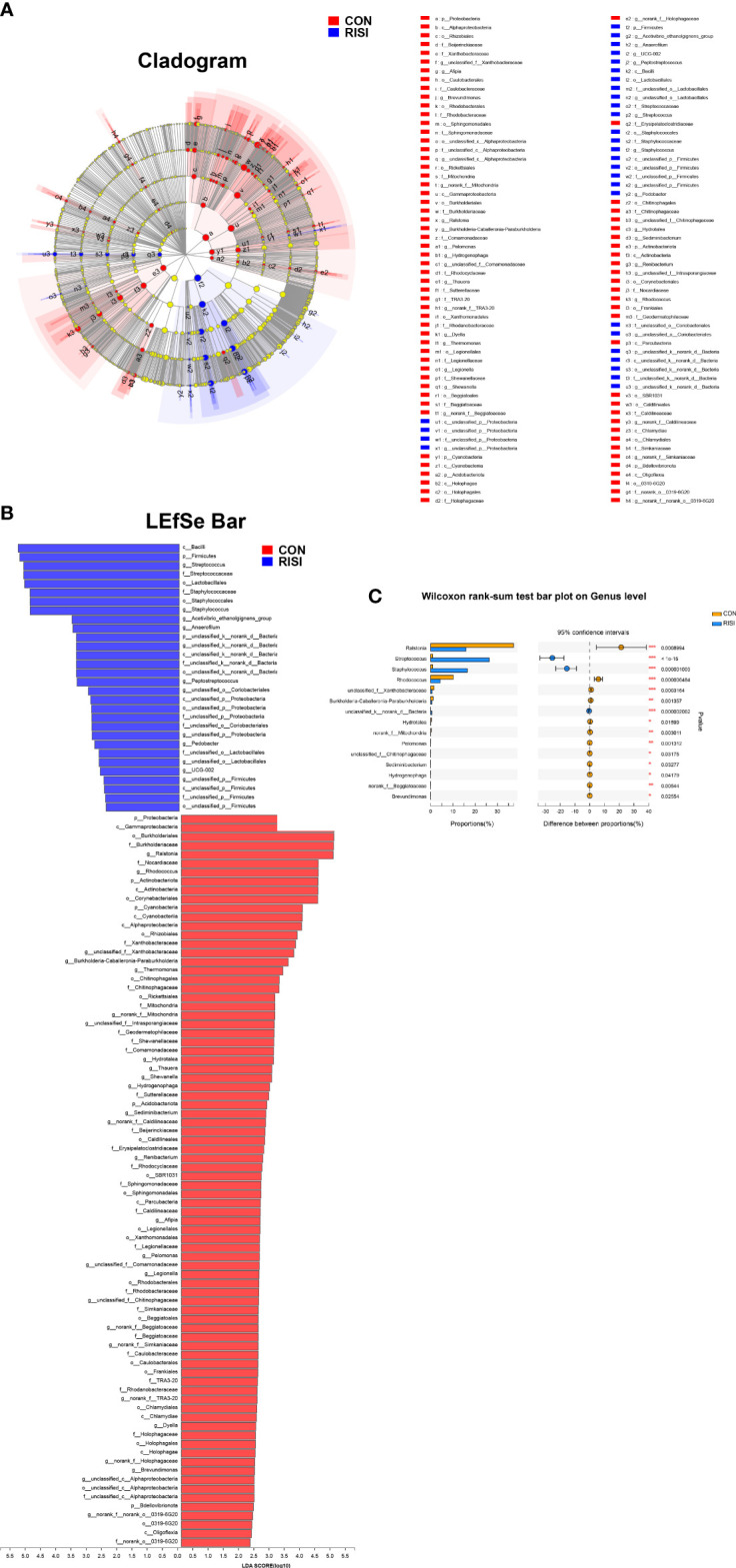
Results of linear discriminant analysis (LDA) effect size (LEfSe). **(A)** Hierarchical tree map of multilevel species. **(B)** LDA histogram showing the microbial groups that play significant roles in several groups. The LDA scores obtained by linear regression analysis showed that the higher the score, the greater the effect of species abundance on the difference. **(C)** Relative abundance of the most predominant genera in cutaneous microbiomes of radiation-induced skin injury (RISI) and healthy controls.

LEfSe was used to discover high-dimensional biomarkers and determine their genomic characteristics (**Figures 3A, B**). LDA can estimate the effect of abundance of each component (species) on the difference effect. Phylum-level analyses of the RISI microbiomes revealed the significant effect of Firmicutes (LDA = 5.223, *p* = 2.42E−5) on RISI, while order-level analyses revealed that Lactobacillales (LDA = 5.07, *p* = 7.79E−06) and Staphylococcales (LDA = 4.89, *p* = 2.75E−06) were the significant orders. We did a further analysis to determine the core species that play key roles in Firmicutes. The results identified *Streptococcus* (genus of Streptococcaceae; LDA = 5.10, *p* = 1.0E−07), *Staphylococcus* (genus of Staphylococcaceae; LDA = 4.89, *p* = 1.0E−06), *Acetivibrio ethanolgignens group* (genus of Lachnosiraceae; LDA = 3.51, *p* = 0.02), *Peptostreptococcus* (genus of *Peptostreptococcaceae*; LDA = 3.35, *p* = 0.04), *Anaerofilum* (genus of *Ruminococcaceae*; LDA = 3.48, *p* = 0.04), and *UCG-002* (genus of Oscillospiraceae; LDA = 2.59, *p* = 0.03) as the core genera of Firmicutes. In addition, *Ralstonia* (genus of Burkholderiaceae) was significantly decreased in RISI (LDA = 4.98, *p* = 8.50E−04) (**Figure 3C**).

### Interaction network analysis

**Figure 4:**
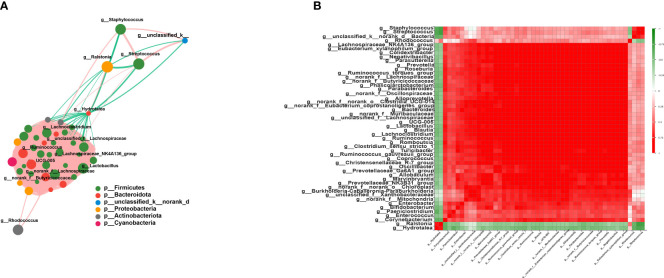
Interaction network and correlation analysis. **(A)** Interaction network of the top 50 species in total abundance. The *size of the nodes* represents the abundance of species, while *different colors* represent different species. The *color of the connecting lines* represents positive and negative correlations: *red lines* denote positive correlation, while *green lines* indicate negative correlation. The *thickness of the line* represents the magnitude of the correlation coefficient. **(B)** Correlation coefficient heatmap. Both the *x*- and *y*-axes indicate the species, with *red* denoting positive correlation and *green* indicating negative correlation.

As expected, the members of Firmicutes had the closest interaction with other species in the network. Among these, multiple members of Lachnospiraceae, such as *unclassified_f_Lachnospiraceae*, *norank_f_Lachnospiraceae*, *Lachnoclostridium*, *Blautia*, and *Lachnospiraceae_NK4A136_group*, were found positively correlated with other Firmicutes species (*r* > 0.6, *p* < 0.05) (**Figure 4B**). Additionally, Streptococcaceae and *Staphylococcus* showed a close correlation with each other (*r* = 0.79, *p* = 2.45E−13) (**Figure 4B**). *Lactobacillus* also occupied an important position in this network, and the average correlation coefficient between *Lactobacillus* and the members of Lachnospiraceae was greater than 0.9 (**Figure 4A**). Moreover, *Hydrotalea* and *Ralstonia* showed a significant correlation with each other (*r* = 0.74, *p* = 2.8E−11), but were negatively correlated with members of Firmicutes.

### Function prediction of cutaneous microbiomes

**Figure 5:**
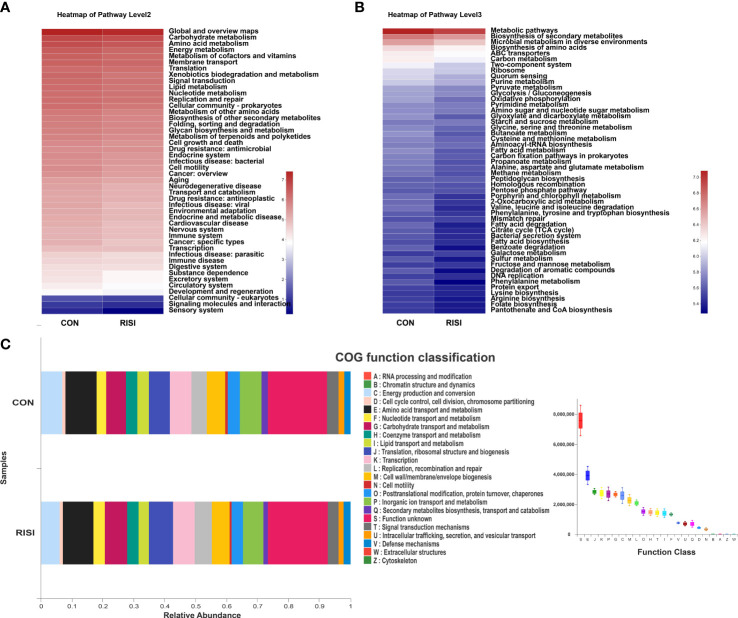
Differentially abundant metabolic pathways between radiation-induced skin injury (RISI) and healthy controls. **(A)** Kyoto Encyclopedia of Genes and Genomes (KEGG) pathways at level 2. **(B)** KEGG pathways at level 3. **(C)** Clusters of Orthologous Genes (COG) function.

The overall function of cutaneous microbiomes may be related to the pathogenesis of RISI, providing a number of potential targets for its prevention and treatment. The differential metabolic pathways were mainly associated with carbohydrate metabolism [butanoate, glyoxylate, dicarboxylate, and propanoate metabolism and citrate cycle—tricarboxylic acid (TCA) cycle], amino acid metabolism (phenylalanine, tryptophan, and histidine metabolism), energy metabolism (oxidative phosphorylation, sulfur metabolism, and carbon fixation pathways in prokaryotes), and lipid metabolism (fatty acid degradation and biosynthesis). In addition, membrane transport [ATP-binding cassette (ABC) transporters], nucleotide metabolism (purine metabolism), endocrine system [peroxisome proliferator-activated receptor (PPAR) signaling pathway], and cell motility (bacterial chemotaxis) may contribute to the progression of RISI (**Figures 5A–C**).

### Alterations in clinical patients

**Figure 6:**
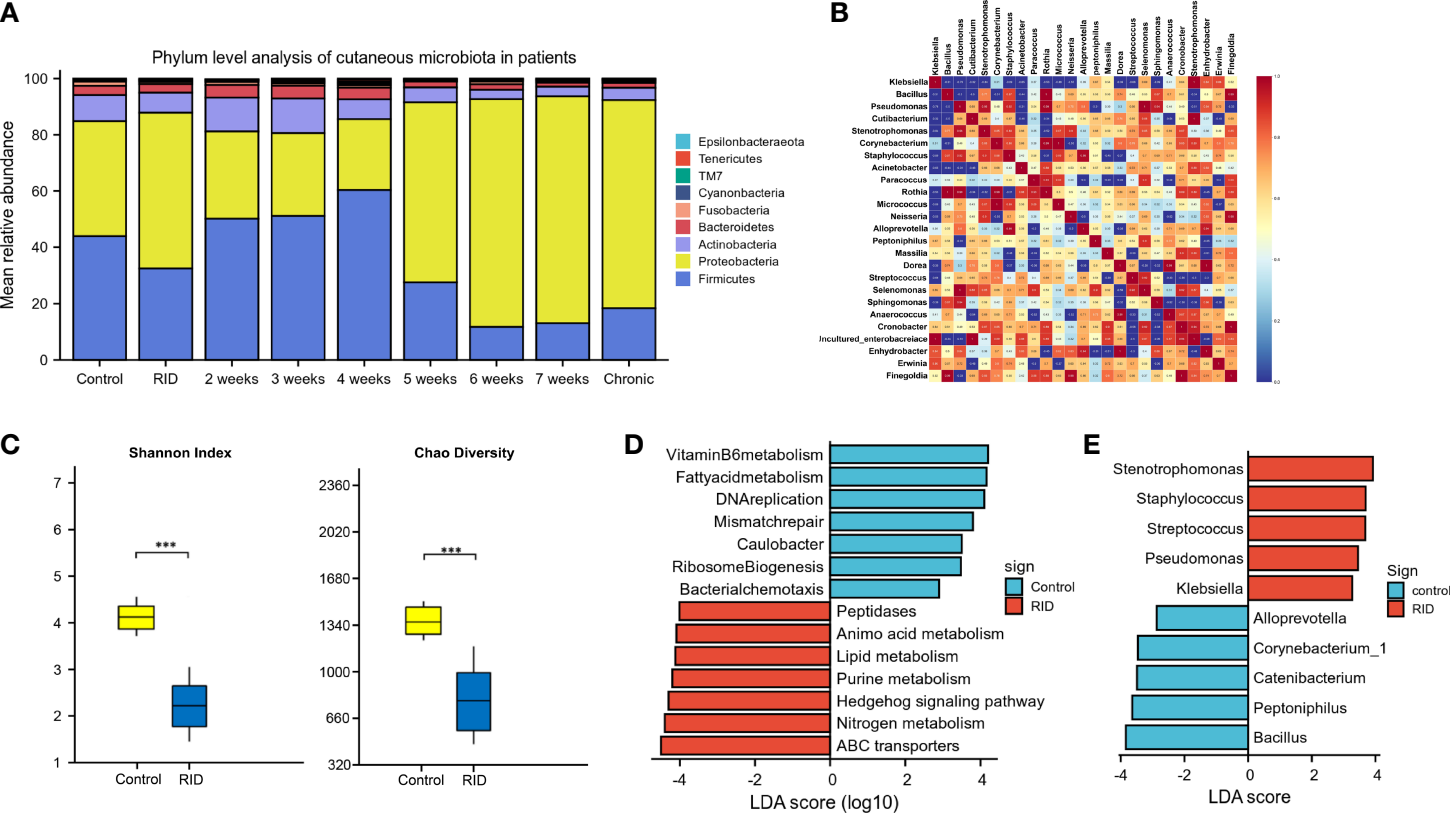
Alterations in patients with radiotherapy-induced dermatitis. **(A)** Changes in the microbial structure of patients with radiation-induced dermatitis (RID) with different prognosis. **(B)** Heatmap of the correlation coefficients. **(C)** Change of species diversity. **(D)** Differential metabolic pathways. **(E)** Linear discriminant analysis effect size (LEfSe) in patients with radiation-induced skin injury (RISI) compared with healthy subjects. *p < 0.05; **p < 0.01; ***p < 0.001.

According to the patient data from BioProject 665254, the adverse effects of undergoing radiotherapy can lead to significant changes in the skin microbiomes (**Figure 6**). Overall, radiotherapy induced a significant reduction in bacterial diversity (Shannon and Chao: *p* < 0.001) (**Figure 6C**). The microbiome associated with the rapid healing of RISI (2–4 weeks) was significantly related to the predominance of Firmicutes (average relative abundance = 52%; Kruskal–Wallis: *p* = 5.7E−4) (**Figure 6A**). In contrast, the microbiota of chronic ulcers was clearly dominated by Proteobacteria and the low abundance of Firmicutes (average relative abundance = 74% and 18%, respectively; Kruskal–Wallis: *p* = 8.3E−4). LEfSe revealed the significant enrichment of *Klebsiella* (LDA = 3.27, *p* = 0.0078), *Pseudomonas* (LDA = 3.45, *p* = 6.23E-04), and *Staphylococcus* (LDA = 3.69, *p* = 0.0004) in RISI compared with healthy subjects (**Figure 6E**).

The differential metabolic pathways were mainly associated with epidermal integrity, alteration in pH, lipid metabolism (fatty acid metabolism), amino acid metabolism, histidine metabolism, membrane transport (ABC transporters), Hedgehog signaling pathway, nitrogen metabolism, and peptidases (**Figure 6D**).

## Discussion

With the wide application of radiological instruments and radioactive materials in medicine and industry, radiation-induced injury is still a serious concern in nuclear safety (Rios et al., 2020; Ashack et al., 2020). RISI, the most common type of injury, has progressed to some form of radio lesions, including erythema, desquamation, ulceration, and cutaneous tumor. According to statistics, almost 50% of patients eventually died of skin wound events from the Chernobyl nuclear accident (Steinert et al., 2003). RISI can seriously affect the quality of life of patients. It represents a challenge for clinicians and is regarded as an additional burden in the healthcare expenditure (Yao et al., 2021; Xie et al., 2021; Hao et al., 2022). However, the exact pathogenic molecular mechanism of RISI is still unclear.

The skin microbiota itself is a barrier against environmental invasion (radiation, climate, or pollution) and infection by multiple pathogenic microbes (Uberoi et al., 2021; Harris-Tryon and Grice, 2022). The culture-based approaches used in the clinic have not been able to determine the integral role of the entire community. Therefore, the application of culture-independent techniques, such as metagenomics and 16S rRNA, to characterize microbial communities provides important new insights into the diversity of the microbial world and its role in dermatological health (Yadav et al., 2022; Liu et al., 2022).

Disruption to the structural integrity of skin microbes is often associated with the progress of disease (Byrd et al., 2017). It has been reported that the alpha diversity was significantly reduced in dermatological disorders such as atopic dermatitis and psoriasis (Ramadan et al., 2019; Elsherbiny et al., 2020). In our animal study, the differences in the alpha diversity indices were not significant between the acute radiation injury and control groups. Subsequent data analysis of clinical patients found that radiotherapy induced a significant reduction in the bacterial diversity (Shannon and Chao: *p* < 0.001). This result was due to a large proportion of the clinical cohort comprising patients with chronic ulcers and lesions with delayed healing whose microbiome structure has been severely damaged. This is similar to the results showing that radiotherapy can significantly reduce the diversity of the digestive tract microbiome (Kumpitsch et al., 2020). The pathophysiology mechanism of RISI was mainly associated with epidermal disturbances, fibrosis, vascular injury, ROS damage, and immune disorder, leading to the lack of oxygen and nutrients in the skin. These factors explain the radiation-induced reduction in microbial diversity. In addition, the injury recovery process can be accelerated by activating the aryl hydrocarbon receptor (AhR) in keratinocytes and restoring the skin microbiome on the wound (Uberoi et al., 2021; Marvasi et al., 2022). This reflects the protective role of cutaneous microbiota in the maintenance of the epidermis.

Taxonomic analysis of the skin microbiota associated with acute radiation injury highlighted the high prevalence of Firmicutes. The results of clinical patients revealed that rapid healing of RISI (2–4 weeks) was significantly accompanied by the predominance of Firmicutes (abundance = 52.2%). In contrast, chronic ulcers were significantly related to the low abundance of Firmicutes (18.36%). It has been reported that Firmicutes can mediate intestinal radioprotection in mice by alleviating the effects of DNA damage. The expression levels of the DNA damage-related markers (gH2AX, p53, and 53BP1) were reduced in the Firmicutes- and short-chain fatty acid (SCFA)-treated groups compared with the controls. We speculated that the predominance of Firmicutes is a stress response of the skin flora to resist external radiation injury. Several members of the Firmicutes phylum are probiotics that can resist dehydration and extreme environments (Wozniak et al., 2022). When bacteria (*Lactobacillus* and *Lachnospira*) ferment carbohydrates, they produce metabolites, including vitamins and SCFAs such as butyrate and lactate (Li et al., 2022). Butyrate helps prevent inflammation and maintains the intestinal epithelial stability. *Lactobacillus* and its lysate reduce the pro-inflammatory cytokines interleukin 6 (IL-6) and IL-8 and enhance the levels of laminin A/B in the human epidermis, suggesting a positive impact on the skin barrier (Khmaladze et al., 2019). Therefore, we regard Firmicutes as the core phylum of early radiation protection.

LEfSe identified several species that have a significant impact on RISI. *Streptococcus* is a genus of Streptococcaceae belonging to the order Lactobacillales. According to relevant reports, *Streptococcus salivarius K12* can alleviate radiation-induced oral mucositis in mice by decreasing the abundance of oral anaerobes, inhibiting NI1060 in *Pasteurella* and downregulating the expression of nitrate reductase (Wang et al., 2021). *Staphylococcus* is a member of the healthy skin microbiota, but it can also cause disease (Williams et al., 2021). Although *Staphylococcus* was significantly enriched in RISI microbiomes, it was overrepresented in patients with rapidly healed RISI. This is consistent with the results of our animal experiments in which the stress of acute radiation injury induced a high abundance of *Staphylococcus.* There is increasing evidence that *Staphylococcus epidermidis* downregulates the pro-inflammatory cytokine IL-6 *via* its metabolite butyric acid and the SCFA receptor (Keshari et al., 2019). Peptides and other metabolites produced by *S. epidermidis* might also contribute to normal defense on the surface of human skin (Cogen et al., 2010; Oliveira et al., 2021). Additionally, previous studies have reported that *Staphylococcus aureus* infection is associated with limited exacerbation and short hospital stay (Armbruster et al., 2016). One of the interesting findings is the significant positive association between *Streptococcus* and *Staphylococcus* in RISI (*r* = 0.79, *p* = 2.45E−13). Notably, *Klebsiella* was considered as a core species that was significantly overrepresented in RISI patients with chronic ulcer and diabetes. However, both of *Streptococcus* (*r* = −0.66, *p* < 0.05) and *Staphylococcus* (*r* = −0.89, *p* < 0.05) were significantly negatively correlated with *Klebsiella.* These observations matched the protective roles of probiotics by secreting antimicrobials and metabolites that resist pathogenic microbial invasion and maintain epidermal integrity (Kazemi et al., 2022). It must be pointed out that several risk factors can influence the incidence and severity of RISI. These risk factors may be intrinsic, including age, ethnicity, gender, malnutrition, location and stage of the tumor, and concomitant diseases such as systemic inflammation and diabetes mellitus. In our study, some of the included clinical patients had systemic diseases such as diabetes, which affected the process of RISI development and healing and the skin microbiota.

In the univariate network analysis, Lachnospiraceae and *Lactobacillus* occupied important positions and were positively correlated with multiple members of Firmicutes. Lachnospiraceae is known for its ability to synthesize SCFAs through the fermentation of dietary carbohydrates (Hexun et al., 2022). SCFAs are crucial substrates of the skin microbiota and epithelium cells that could maintain the acid–base balance, inhibit the growth of harmful pathogens, and regulate the immune system and inflammatory responses (Arpaia et al., 2013; Agus et al., 2021). *Lactobacillus* can ferment carbohydrates to lactic acid, which can also be regarded as a type of SCFA. By investigating the gut microbiome of mice that survived a high dose of radiation, Lachnospiraceae and Enterococcaceae were found to be the most enriched bacteria in these elite survivors (Guo et al., 2020). After bacterial reconstitution, the grouped mice received a high-dose radiation. Mice inoculated with Lachnospiraceae showed the greatest improvement in survival rate and clinical score, while those given *Lactobacillus rhamnosus* showed a 40%–60% increase in the survival rate.

The differential metabolic pathways of RISI were mainly associated with carbohydrate metabolism (butanoate and propanoate metabolism), amino acid metabolism (tryptophan and histidine metabolism), energy metabolism, endocrine system (PPAR signaling pathway), and lipid metabolism (fatty acid degradation and biosynthesis). An untargeted metabolomics study revealed that tryptophan metabolism selectively increased in the elite survivors affected by radiation (Guo et al., 2020). It was also found that tryptophan metabolites, including I3A and KYNA, significantly increased the survival rates and decreased the clinical scores of radiation-treated mice. Butyrate and propionate mediate radioprotection by alleviating the levels of the DNA damage-related proteins and reducing the ROS levels by 50%–60% (Guo et al., 2020; Liu et al., 2021). Interestingly, a report illustrated the effect of ionizing radiation on skin lipid metabolism. The function of adipose tissue has traditionally been understood as energy storage, physical buffering, temperature regulation, and thermal insulation (Wrba et al., 2022). Xiao et al. revealed that radiation modulates the skin lipid mass and profiles and downregulates multiple lipid metabolism pathways, including PPAR signaling. Rats fed with a high-fat diet with increased fat accumulation were found resistant to RISI (Xiao et al., 2020). It has been shown that PPAR alpha (PPARα) activation can significantly ameliorate RISI (Liu et al., 2022). PPARα is a member of the PPAR nuclear hormone receptor superfamily, which can be activated by a variety of ligands including fatty acids (Bougarne et al., 2018; Takada and Makishima, 2020). It has been reported that SCFAs can induce the expression of PPARα in a time- and concentration-dependent manner in the intestinal epithelial cell line (Higashimura et al., 2015). This is a potential molecular mechanism of radiation protection mediated by cutaneous microbiomes, which influence skin metabolism through their metabolite fatty acids and SCFAs.

This study has some limitations. Firstly, we focused solely on the unculturable approach. In further mechanism research, the culturable approach should also be combined to isolate the probiotics and causative agents in order to define their virulence characteristics, including the antimicrobial resistance patterns. In addition, there is still a lack of microbiome characterization at different sampling times. Moreover, microbial communities in the human skin are distinct and significantly less diverse than those in animal models. To better understand the mechanism of RISI, we combined the results from animal models and human data to identify some common changes. This study provides data on potential core microbes and metabolic pathways for further mechanism research and treatment development. However, further functional validation is required to elucidate their roles in RISI.

## Conclusion

We have investigated the alterations and functions of cutaneous microbiomes mediated by RISI. Our study provides new insights into the potential mechanism and microbial changes in the progression of RISI. The overwhelming predominance of members of Firmicutes, such as Streptococcaceae, Staphylococcaceae, Lachnospiraceae, and *Lactobacillus*, potentially help promote rapid healing in RISI. The microbiota–metabolite axis plays a critical role in RISI and provides promising therapeutic targets for the treatment of adverse side effects.

## Data availability statement

The datasets presented in this study can be found in online repositories. The names of the repository/repositories and accession number(s) can be found in the article/**Supplementary Material**.

## Ethics statement

The animal study was reviewed and approved by the Animal Experimentation Ethics Committee at China National Nuclear Corporation 416 Hospital. Written informed consent was obtained from the individual(s) for the publication of any potentially identifiable images or data included in this article.

## Author contributions

BH designed and carried out the bioinformatics analyses and animal experiment and drafted the manuscript. LA, WS, and TY helped with drawing the figures and with the animal experiment. D-JY and HZ initiated the study. All authors contributed to the article and approved the submitted version.

## Funding

This work was supported by China National Nuclear Corporation Medical Department “Nuclear Medicine Technology Innovation” Project (ZHYLYB2021009) and the National Natural Science Foundation of China (32071238 and 82073477). The Fundamental ResearchFunds for the Central Universities andYoung Talent Program of China National Nuclear Corporation (CNNC2021136). TheScience and Technology Program of Suzhou (SKY2021007) , Discipline Construction of The Second Affiliated Hospital of Soochow University (XKTJ-TD202001).

## Conflict of interest

BH, WS, and D-JY were employed by China National Nuclear Corporation 416 Hospital.

The remaining authors declare that the research was conducted in the absence of any commercial or financial relationships that could be construed as a potential conflict of interest.

## Publisher’s note

All claims expressed in this article are solely those of the authors and do not necessarily represent those of their affiliated organizations, or those of the publisher, the editors and the reviewers. Any product that may be evaluated in this article, or claim that may be made by its manufacturer, is not guaranteed or endorsed by the publisher.
